# Fecal microbiota transplantation improves hepatic steatosis induced by HFD in a mouse model associated with liver ILC1 regulation and indole-3-carbinol level

**DOI:** 10.3389/fnut.2025.1500293

**Published:** 2025-02-19

**Authors:** Peng-fei Hou, Yu Yao, Ying Wu, Hong-tao Yu, Yu Qin, Long Yi, Man-tian Mi

**Affiliations:** ^1^Research Center for Nutrition and Food Safety, Chongqing Key Laboratory of Nutrition and Health, Chongqing Medical Nutrition Research Center, Institute of Military Preventive Medicine, Third Military Medical University, Chongqing, China; ^2^Department of Nutrition, The Sixth Medical Center of Chinese People’s Liberation Army General Hospital, Beijing, China

**Keywords:** metabolic dysfunction-associated steatotic liver disease, liver ILC1, fecal microbiota transplantation, Indole-3-carbinol, aryl hydrocarbon receptor, high-fat diet

## Abstract

**Background:**

The prevalence of metabolic dysfunction-associated steatotic liver disease (MASLD) has increased worldwide. In recent years, fecal microbiota transplantation (FMT) has become an important promising method for the treatment of MASLD. However, the mechanism remains unclear.

**Methods:**

The animal model with C57BL/6 male mice induced by high-fat diet (HFD) for 12 weeks has been introduced. Fecal microbiota and indole-3-carbinol (I3C) was given by oral gavage.

**Results:**

Our study demonstrated that a 6-week healthy gut microbiota transplantation tended to ameliorate hepatic steatosis and reverse the decreased liver ILC1 induced by HFD. Interestingly, there was also a negative correlation between liver ILC1 and liver TG, TC level. Furthermore, the protective effect was associated with the elevated levels of serum indole-3-carbinol (I3C). Also, a I3C administration for 6 weeks improved liver steatosis and increased the frequency of liver ILC1 induced by HFD through aryl hydrocarbon receptor (AhR) activation. Moreover, I3C binds to the residues of ALA349, PHE348, LEU309, TYR316, PHE318 on AhR through hydrogen bonds, *Π* bonds, hydrophobic bonds which was proved by molecular docking.

**Conclusion:**

To conclude, our data demonstrated that FMT improved liver steatosis induced by HFD associated with liver ILC1 regulation and indole-3-carbinol level. The study highlighted the potential treatment value of FMT and microbiota-derived I3C in the MASLD treatment and regulation of liver ILC1 function.

## 1 Introduction

Non-alcoholic fatty liver disease (NAFLD), also known as metabolic dysfunction-associated steatotic liver disease (MASLD), is known to be characterized by the presence of micro- or macrovesicles in more than 5% of hepatocytes (1). Recently, the new definition and underlying pathophysiology emphasized the important role of metabolic disorder in the occurrence of MASLD, especially lipid homeostasis imbalance (2). MASLD has emerged as the most prevalent liver disease and a significant public health concern, with an estimated overall prevalence of approximately 32.4% worldwide (3), while a higher prevalence of 44.39% in China (4). However, there are currently no approved pharmacological treatments available.

In several studies, MASLD patients showed significant gut microbiota dysbiosis, which is closely related to the occurrence of metabolic syndrome (5). Meanwhile, fecal microbiota transplantation (FMT) from MASLD patients can trigger hepatic steatosis and lipid accumulation in mice (6). Interestingly, researchers found that FMT from the healthy donors could reduce body weight, fasting blood glucose, total cholesterol, triglycerides, low-density lipoprotein, and systemic endotoxin levels in db/db mice model, and improved insulin resistance and liver lipid deposition (7–9). Thus, FMT from the healthy donors may be a potential treatment option for MASLD patients. But these studies also have some limitations and the mechanism of FMT has not been fully elucidated. Previous studies demonstrate that microbiota-derived metabolites such as indole derivatives might be the key mechanism for the biological effects of gut microbiota in the lipid metabolism regulation (10). Indole-3-carbinol (I3C) is not only a dietary compound which was found in cruciferous vegetables (11), but also produced by gut microbiota such as *Limosilactobacillus reuteri WX-94* and *Lactobacillus plantarum FRT4* as an indole metabolite (12, 13). I3C has gained considerable attention as promising preventive and treatment agents for various diseases such as cancer, diabetes and obesity (14–16). The aryl hydrocarbon receptor (AhR) is a basic helix–loop–helix transcription factor which play a critical role in the regulation of immune cell differentiation when ligated by an AhR ligand (17, 18). CH-221391 was an AhR antagonist as reported previously. As for the mechanism of I3C, being an AhR ligand, I3C supplementation could activate AhR and reduce the associated immunopathology (19). But the studies have mainly focused on parenchymal cells such as hepatocytes, rather than non-parenchymal cells (NPCs) such as liver reginal immune cells.

Recent research has shed light on the concept of immune metabolism, introduced by Mathis and Shoelson, which emphasizes the role of tissue reginal immune cells in the regulation of metabolism homeostasis (20). The liver proved to be not only the most important metabolic organ, but also a well-recognized and complex immunological organ that contains numerous adaptive and innate immune cells (21, 22). Emerging evidences indicate that liver reginal immune cells play critical and different roles in the lipid metabolism disorder and the MASLD occurrence as well as the progression of metabolic dysfunction-associated steatohepatitis (MASH) (23, 24). Innate lymphoid cells (ILCs) are newly described NPCs in liver, which are heterogeneous population of non-B non-T lymphocytes originating from common lymphoid progenitors but lacking antigen-specific receptors (25–27). Liver ILC1s, a newly defined liver reginal immune cell, was revealed to play an essential role in liver virus infection, tumor development, liver injury and regeneration (28–31). Recently, we found that liver ILC1s play a key role in the regulation of liver lipid metabolism. However, it was unclear whether FMT of the healthy donors improved hepatic steatosis was associated with liver ILC1 function regulation or not. And if so, further additional studies are to define the underlying mechanisms in this respect.

Therefore, this study, based on the established high-fat diet (HFD)-induced hepatic steatosis of the mice model, aimed to clarify the effect and mechanism of FMT from heathy donors improved hepatic steatosis and regulated the liver ILC1 function. The study might help us to sufficiently understand the mechanism involved in the benefits of FMT from healthy donors on hepatic steatosis and liver ILC1 function regulation, and also provide the theoretical basis for the FMT as an ideal therapeutic approach to be applied in MASLD patients.

## 2 Materials and methods

### 2.1 Animal experiment

Male C57BL/6 mice weighing 19 ~ 21 g at 6–8 weeks of age were obtained from the center of experimental animals of the Army Medical University and bred in a controlled environment under 22–25°C and humidity of 50 ± 5% on a 12 h light–dark cycle. Food and water were changed every 3 days and were provided ad libitum. The mice were allowed 1 week to adapt to the laboratory environment and the study contained three experiments:

**Experiment #1**. The mice were randomly divided into the three study groups, i.e., (1) Normal control diet group (CON), which was administrated the control diet (10% fat, 70% carbohydrate, 20% protein; XTCON50J; Jiangsu Xietong Pharmaceutical Bio-engineering Co., Ltd., China; *n* = 6); (2) High-fat diet group (HF60), which was administrated the high fat diet (60% fat, 20% carbohydrate, 20% protein; XTCON50J; Jiangsu Xietong Pharmaceutical Bio-engineering Co., Ltd., China; *n* = 6); (3) Fecal microbiota transplantation group (FMT; *n* = 6). Both HF60 and FMT groups were administered a high-fat diet for 12 weeks. In the FMT group, fecal samples were collected from the Con group mice. These feces were collected daily, homogenized, and suspended in 0.9% saline solution (50 mg/mL) (26). Following centrifugation at 3000 rpm for 5 min, each recipient mouse received 250 μL/day fecal microbiota suspension through intragastric administration for 6 weeks from the sixth week of high fat diet fed mice. The dosage of FMT was as reported previously (32). The HF60 group was administered the equal amount of normal saline.

**Experiment #2**. The mice were randomly divided into the two study groups, i.e., (1) High-fat diet group (HF60; *n* = 6); (2) Indole-3-carbinol administration group (I3C; *n* = 6). Both groups have been administered the high-fat diet for 12 weeks. Mice in I3C group were given I3C (Sigma-Aldrich, Germany) by gavage daily in the dosage of 50 mg/kg/day which was dissolved into a suspension in 0.9% saline for 6 weeks from the sixth week of high fat diet fed mice. The HF60 group was given similar amount of normal saline.

**Experiment #3**. The mice were randomly divided into the two study groups, i.e., (1) HFD with CH-221391 group (HF60 + CH; *n* = 6); (2) I3C administration of CH-221391 group (I3C + CH; *n* = 6). Both groups were administered HFD for 12 weeks, and received CH-221391 (Selleck, United States) via intraperitoneal injection in a dosage of 10 mg/kg/day for 4 weeks from the ninth week. Meanwhile, I3C + CH group was given I3C daily as previously shown. And the dosage for AhR antagonist CH-221391 was as reported previously (33).

The body weight and food consumption were recorded on a weekly basis. Body composition detection was performed at the 12 week after high-fat diet fed. Upon decapitation, the serum was prepared by solidification and centrifugation (4°C, 3500 rpm, 15 min) and then stored at −80°C. Liver and adipose tissues were collected and stored at −80°C for biomedical analysis. The animal procedures were approved by the Animal Ethics Committee of the Third Military Medical University (AMUWEC20211120).

### 2.2 Body composition detection

According to the operating instructions of the Small Animal Body Composition Analyzer (QMR06-090H-PRO, Suzhou Newman Analytical Instruments Co., Ltd., China), the body composition of mice was detected.

### 2.3 Biochemical parameters

The total triglycerides and total cholesterol level of liver samples were determined using the enzymatic assay kit (Solarbio, Beijing, China). The free fatty acid level in serum samples was determined applying an enzymatic assay kit (Solarbio, Beijing, China). The serum I3C content was determined using an enzyme-linked immunoassay kit (Ao Ruida Bio, Guangzhou, China). The aspartate aminotransferase; alanine aminotransferase; were performed with the Automatic Analyzer 3,110 (Shanghai, China) according to the operating instructions.

### 2.4 Quantitative real-time polymerase chain reaction

The total RNA was extracted with TRIzol reagent (ABclonal, China). QTower 2.2 real-time PCR system (Analytik Jena, Germany) was used to perform the qRT-PCR with SYBR Premix Ex Taq II (ABclonal, China). All primers were listed in Table 1. The relative mRNA expression levels of the genes were normalized down to that of *β*-actin and calculated by the 2 − ΔΔCt.

**Table 1:** Sequences of primers used in qRT-PCR.

Genes	Primers(5′ → 3′)	
	Up	Down
*18 s*	ACGGACCAGAGCGAAAGCAT	TGTCAATCCTGTCCGTGTCC
*Fasn*	TCAATGACATTGCGGCAACC	TTGCTGCACTTCTTGGACAC
*Acaca*	CAGCATCTCTAACTTCCTTCACTCC	ACACGAGCCATTCATTATCACTACG
*Srebf1*	ACCGTGGGCTGAGGAAGGATG	CCAGGTTAGAAGCAGCAAGATGTCC
*ATGL*	CAGAGATGGACTTCGATTCCTT	CAGGTGCTCTAGAATTCGATCT
*Cpt1a*	CACAACAACGGCAGAGCAGAG	ACACCACATAGAGGCAGAAGAGG
*PPARα*	AGTCCATCGGTGAGGAGAGC	TGGAAGCTGGAGAGAGGGTG
*CD36*	GCGACATGATTAATGGCACAGAC	GATCCGAACACAGCGTAGATAGAC
*TNFα*	ATCAGTTCTATGGCCCAGACC	ACTTGGTGGTTTGCTACGAC
*CXCL1*	ACCCGCTCGCTTCTCTGTG	ATTCTTGAGTGTGGCTATGACTTCG
*CCL2*	CTCACCTGCTGCTACTCATTCAC	GTATGTCTGGACCCATTCCTTCTTG
*UCP1*	GGAGGTGTGGCAGTGTTCATTG	GCTTTCTGTGGTGGCTATAACTCTG
*Cidea*	CCGTGTTAAGGAATCTGCTGAGG	GGATGGCTGCTCTTCTGTATCG
*Prdm16*	GCCGTTCAAGTGCCATCTGTG	CCTCGTGTTCGTGCTTCTTCAG
*AhR*	CATCGACATAACGGACGAAATC	CTGTTGCTGTTGCTCTAGTTG
*Cyp1a1*	ACCCTTACAAGTATTTGGTCGT	GTCATCATGGTCATAACGTTGG

18 s, 18S ribosomal RNA; Fasn, Fatty acid synthase; Acaca, Acetyl-CoA carboxylase alpha; Srebf1, Sterol regulatory element binding transcription factor 1; ATGL, adipose triacylglyceride lipase; Cpt1a, carnitine palmitoyl transferase 1a; PPARα, peroxisome proliferators-activated receptors α; CD36, Fatty acid receptor CD36; TNFα, tumor necrosis factor α; CXCL1, Chemokine CXCL1; CCL2, Chemokine CCL2; UCP1, Uncoupling protein 1; Cidea, cell death-inducing DNA fragmentation factor, alpha subunit-like effector A; Prdm16, PR domain containing 16; AhR, Aryl Hydrocarbon Receptor; Cyp1a1, cytochrome P450, family 1, subfamily A, polypeptide 1.

### 2.5 Histological evaluation

After decapitation, the liver and adipose tissue samples were fixed in 4% paraformaldehyde for at least 24 h and then embedded in paraffin wax, followed by sectioning into 5 μm slices. The liver section was subsequently deparaffinized and rehydrated through a xylene and alcohol series, then stained with hematoxylin and eosin (HE) using a standard procedure. For the Oil Red O staining, the liver sample cryo sections were prepared in a cryostat. The cryo sections were stained with pre-warmed oil red O working solution for 30 min. After rinsing with distilled water 3 times, then counterstained with hematoxylin for 3 min and gently washed with distilled water. Representative image was shown.

### 2.6 Molecular docking of I3C and AhR

This experiment was performed mainly by online website Yin Fu science^1^: (1) The compound structure of I3C was purchased from PubChem and ZINC15 databases and converted into 3D structure. (2) The crystal structure of mouse AhR was downloaded from the RCSB-PDB database.^2^ (3) The DOCK 6.9 was used and the docking pocket sites (F281, H285, F289, L302, Y304, L309) of AhR crystal structure were selected according to the study (34).

### 2.7 Isolation of liver lymphocytes

Liver lymphocytes isolation was performed according to the previous report (25). In brief, the liver was perfused and excised in RPMI-1640 culture medium through a 70 μm filter (352,350, BD Falcon, United States) after mashed. Liver homogenates cells were resuspended with a 44% Percoll density gradient medium (17–0891-09, GE Healthcare, United States), overlayed on a 67% Percoll medium, and centrifuged at 2000 × rpm for 20 min at 20°C. Buffy coats were collected and washed with PBS supplemented with 5% fetal calf serum (16,140,089, Thermo Fisher Scientific, United States) and used as liver lymphocytes. Liver ILC1s cells were identified as CD45 + TCR*β*-B220-CD49a + CD49b-.

### 2.8 Flow cytometry

For the surface staining, the isolated liver lymphocytes were stained with anti-mouse CD45 (Biolegend, United States), anti-mouse TCR-β (Biolegend, United States), anti-mouse B220 (Biolegend, United States), anti-mouse NK-1.1 (Biolegend, United States), anti-mouse CD49a (Biolegend, United States), and anti-mouse CD49b (Biolegend, United States). The liver ILC1s were analyzed by flow cytometry (LSR Fortessa X20 cell analyzer, BD Bioscience, United States).

For transcription factor staining, after surface staining, cells were fixed, permeabilized and washed with eBioscience Foxp3/transcription factor staining buffer set (Thermo Fisher Scientific) according to the manufacturer’s instructions, and stained with anti-mouse T-bet (Biolegend, United States) or anti-mouse AhR (Biolegend, United States) and then analyzed by flow cytometry (LSR Fortessa X20 cell analyzer, BD Bioscience, United States). Data were analyzed using FlowJo V10.6.

### 2.9 Statistical analysis

Data analysis was performed applying GraphPad Prism 8.0 (GraphPad Software, Inc., La Jolla, CA, United States). All experimental data were expressed as mean ± SEM values. According to the results of normality test, the analysis differences between groups were determined by Student’s *t*-test (two groups) or Wilcoxon test. Three group comparisons were conducted using analysis of variance (ANOVA) followed by the LSD or SNK post-hoc tests for multiple pair-wise comparisons, respectively. Pearson correlation or spearman rank correlation test was used for correlation analysis. *p*-values less than 0.05 were considered statistically significant. All experiments were repeated two times at least.

## 3 Results

### 3.1 FMT from healthy mice donors improved high-fat diet induced hepatic steatosis

**Figure 1:**
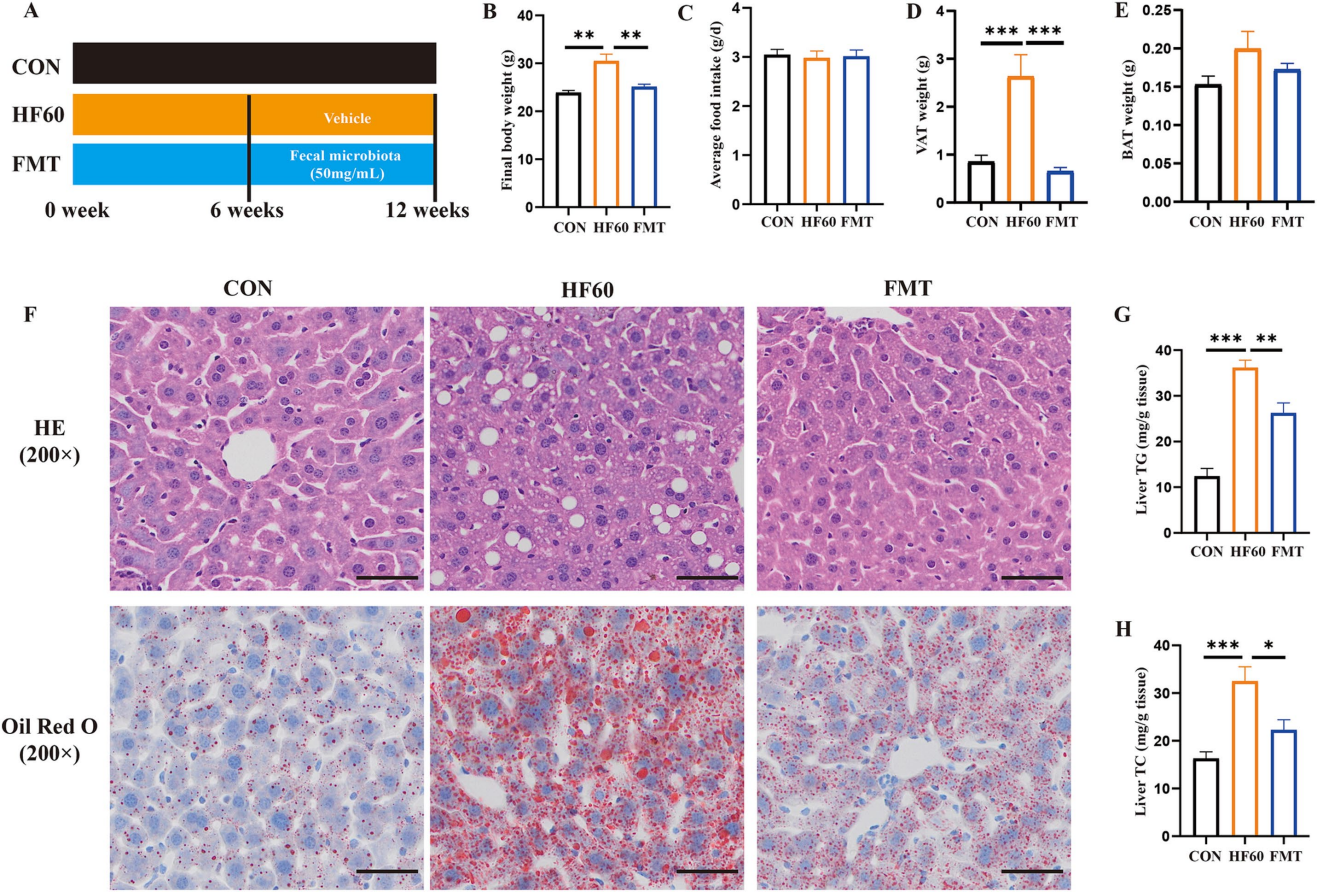
FMT from healthy mice donors improved high-fat diet induced hepatic steatosis. **(A)** Schematic diagram illustrating that 8-week-old male C57BL/6-mice were fed a HFD diet for 12 weeks, and the FMT experiment was performed for 6 weeks with 250 μL/per mice fecal microbiota suspension intragastrically; **(B)** Final body weight of mice; **(C)** Average daily food intake per mouse; **(D)** Weight of visceral adipose tissue (VAT); **(E)** Weight of brown adipose tissue (BAT); **(F)** Representative photographs of the liver sections with H&E (Up), Oil Red O (Down) staining. Scale bar: 50 μm; ×200 magnification. The liver TG **(G)** and TC **(H)** levels were measured with the corresponding assay kits. *p < 0.05, **p < 0.01, ***p < 0.001.

To explore the effect of FMT on HFD-induced hepatic steatosis, the experimental study was performed as shown in Figure 1A. Compared with the CON group mice, the HF60 group mice showed a significant increase of final body weight (Figure 1B), while the average food intake showed no significant changes (Figure 1C). Furthermore, FMT administration significantly reduced the body weight and VAT weight rather than the BAT weight (Figures 1B,D,E). The HE and Oil Red O staining of liver section showed predominantly hepatic steatosis and lipid accumulation characterized as increased large lipid droplets in the HF60 group compared with that in the CON group. Meanwhile, FMT group mice revealed improved liver steatosis compared to the HFD group (Figure 1F). Furthermore, hepatic TG and TC contents were measured and the results revealed FMT significantly reduced the contents of TG and TC in liver (Figures 1G,H). To sum it up, FMT from healthy donors improved high-fat diet induced hepatic steatosis.

### 3.2 FMT from healthy mice donors reversed the decreased liver ILC1 induced by high-fat diet

**Figure 2:**
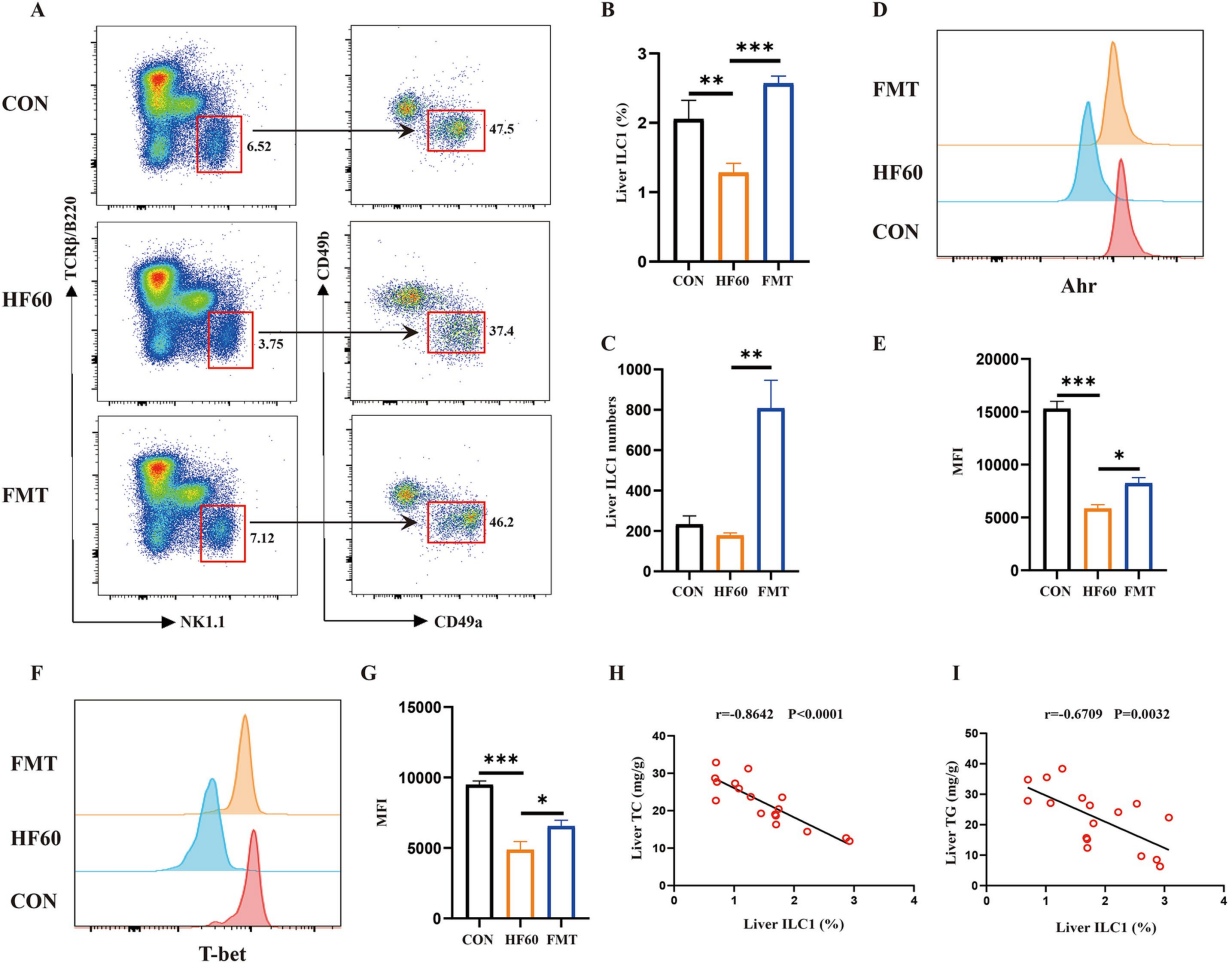
FMT from healthy mice donors reversed the decreased liver ILC1 induced by high-fat diet. **(A)** Representative flow cytometry of liver ILC1 cells (CD45^+^TCRβ^−^B220^−^NK1.1^+^CD49a^+^CD49b^−^); The percentage **(B)** and absolute numbers **(C)** of ILC1 in CD45^+^ lymphocytes; **(D,E)** Representative flow cytometry and MFI of AhR in liver ILC1; **(F,G)** Representative flow cytometry and MFI of T-bet in liver ILC1; Correlation analysis of liver ILC1 frequency with liver TC content **(H)** and liver TG content **(I)**. *p < 0.05, ***p < 0.001.

To clarify the effect of FMT on the regulation of liver ILC1, the liver ILC1 frequency and differentiation associated transcription factors were examined. As compared to the Con group mice, HFD tended to obviously decrease the proportion of the liver ILC1 (Figures 2A,B). And FMT administration could reverse the change of the liver ILC1 proportion and increase the absolute number (Figures 2A–C). We also explored the expression level of liver ILC1 differentiation associated transcription factors T-bet and AhR. The obtained results showed HFD treatment to impair the expression of T-bet and AhR, as indicated the decreased T-bet and AhR MFI (Figures 2D–G). Moreover, it was investigated whether the change of ILC1 frequency was associated with the hepatic steatosis. The results of correlation analysis showed that the liver ILC1 frequency negatively correlated with liver TG and TC content (Figures 2H,I). Altogether, FMT from healthy mice donors could reverse the decreased liver ILC1 induced by HFD and the change of the liver ILC1 frequency was negatively correlated with hepatic steatosis.

### 3.3 FMT from healthy mice donors reversed the decreased liver ILC1 frequency associated with the changes of I3C level

**Figure 3:**
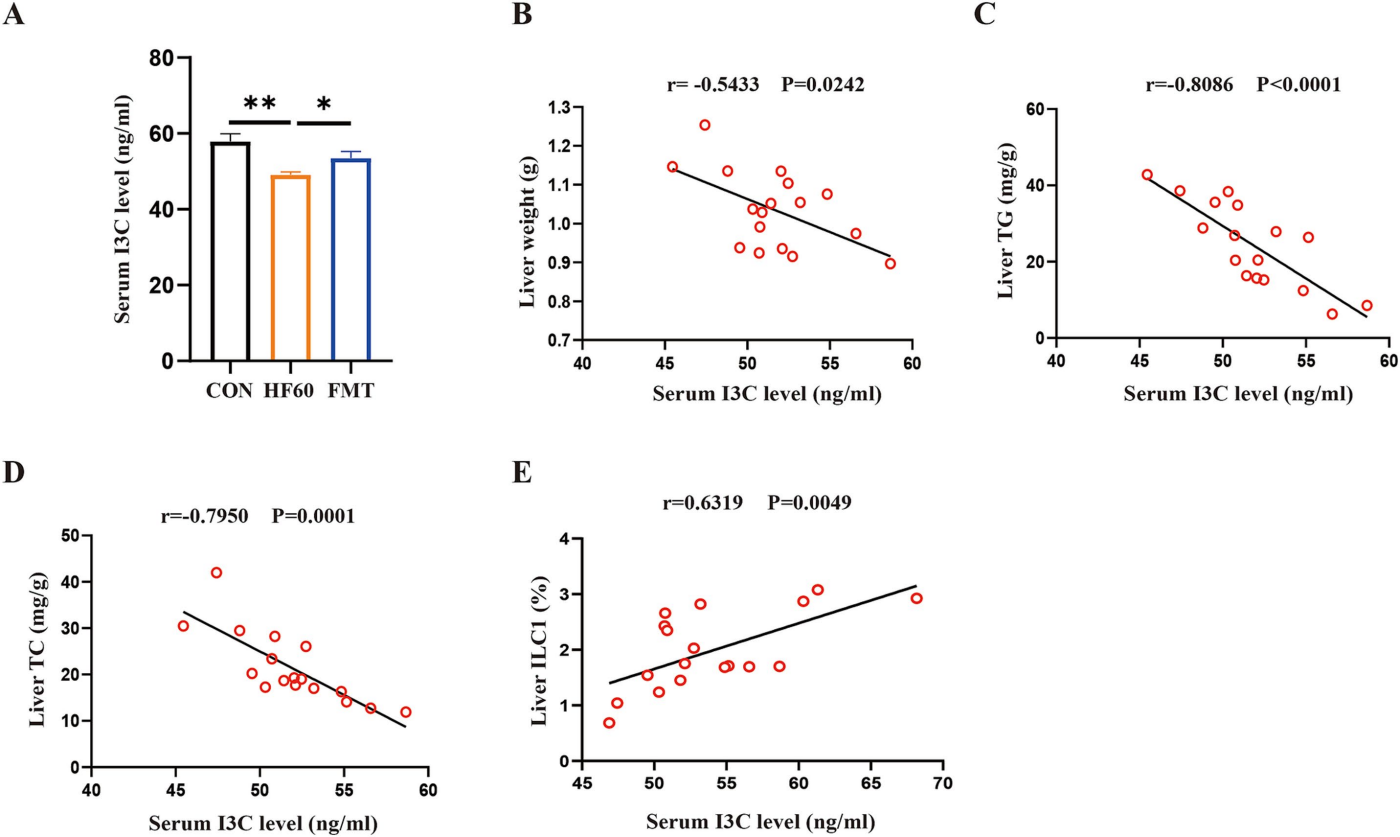
FMT from healthy mice donors reversed the decreased liver ILC1 frequency associated with the changes of I3C level. **(A)** The content of I3C in serum; **(B–E)** Correlation analysis of serum I3C content with liver weight **(B)**, liver TG content **(C)** and liver TC content **(D)** as well as liver ILC1 frequency **(E)**; *p < 0.05, **p < 0.01.

First, the I3C level in all study groups of mice was determined. We found the content of I3C in serum to be obviously decreased in HF60 group, which was increased after FMT administration compared to the HF60 group (Figure 3A). Moreover, the correlation analysis showed that serum I3C content negatively correlated with liver weight, liver TG and liver TC content, correspondingly (Figures 3B–D). Furthermore, the correlation analysis serum I3C level vs. liver ILC1 frequency demonstrated the serum I3C content to positively correlate with liver ILC1 frequency (Figure 3E). Taken together, the serum I3C level was correlated with liver TG and TC content as well as ILC1 frequency in HFD fed or FMT administration mice.

### 3.4 I3C modulates cell expansion and expressions of gene related to adipogenesis, thermogenesis in VAT or BAT induced by HFD

**Figure 4:**
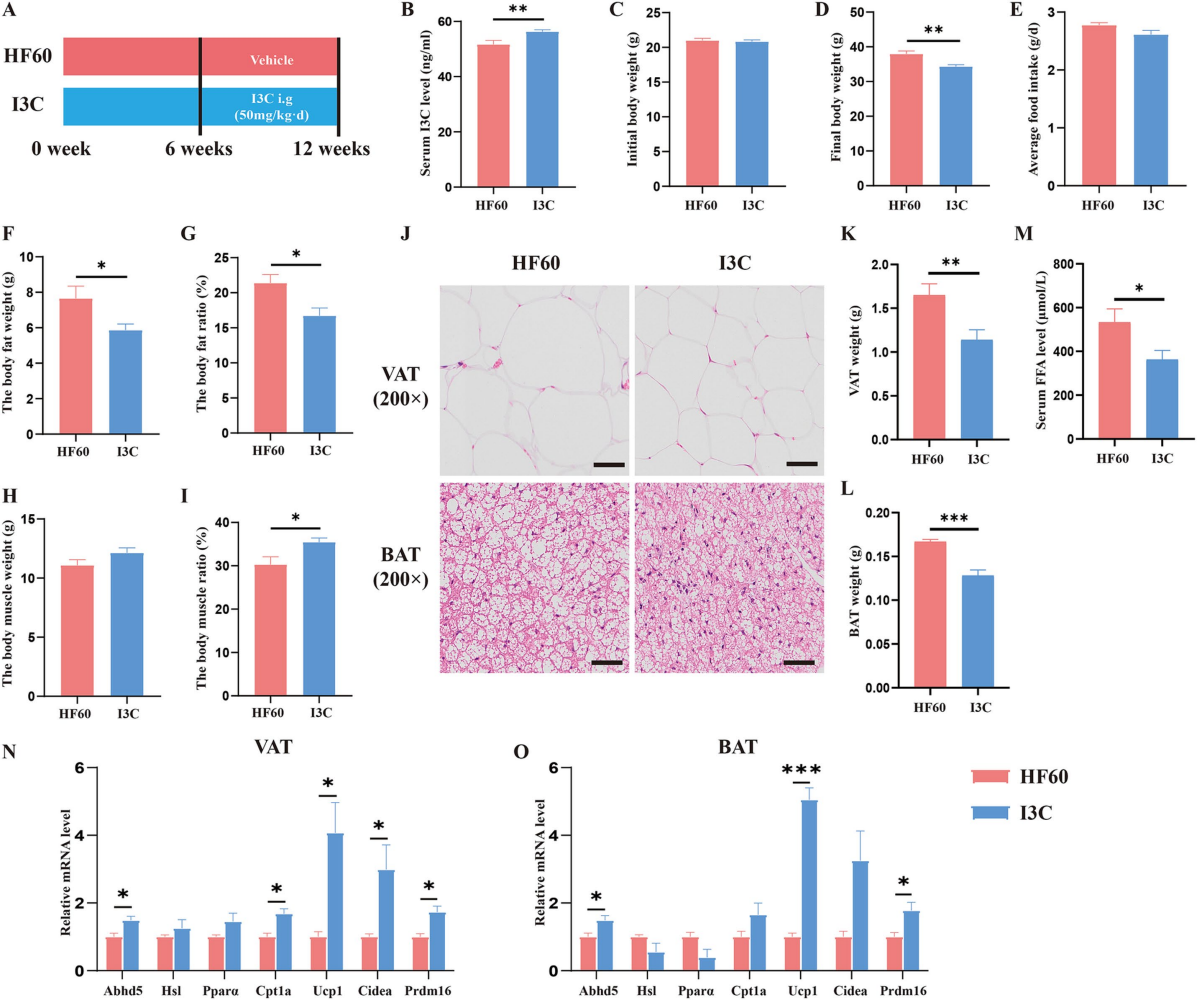
I3C modulates cell expansion and expressions of gene related to adipogenesis, thermogenesis in VAT or BAT induced by HFD. **(A)** Schematic diagram illustrating that 8-week-old male C57BL/6-mice were fed a HFD diet for 12 weeks, and I3C was given with the dose of 50 mg/kg/day intragastrically for 6 weeks; **(B)** The content of I3C in serum; **(C)** Initial body weight of mice; **(D)** Final body weight of mice; **(E)** Average daily food intake per mouse; The body composition of mice included body fat weight **(F)** and body fat ratio **(G)**, body muscle weight **(H)** and body muscle ratio **(I)**; **(J)** Representative photographs of the BAT (Down) and VAT (Up) sections with H&E staining. Scale bar:50 μm; ×200 magnification; **(K)** Weight of VAT; **(L)** Weight of BAT; **(M)** The serum FFA level of mice; The mRNA expressions of the VAT **(N)** and BAT **(O)** which related to lipid metabolism and browning detected by qRT-PCR; *p < 0.05, **p < 0.01, ***p < 0.001.

Base the previously obtained results, we believe the protective effect of FMT to be associated with the I3C level. To clarify the effect of I3C on body composition, schematic diagram of the experiments was shown in Figure 4A. Serum I3C level was increased after I3C administration by oral intake within 6 weeks (Figure 4B). Meanwhile, as expected, the body weight was decreased in I3C treatment group mice (Figures 4C,D), while the average food intake per mice showed no significant difference (Figure 4E). Evaluation of the body composition of the mice showed the body fat weight and body fat ratio to be decreased in I3C group mice, while the body muscle ratio was increased (Figures 4F–I). Furthermore, histological analysis showed that the adipocyte size in VAT was reduced in I3C group mice as compared to the one in the HF60 group mice (Figure 4J). Meanwhile, the fat deposition in BAT was improved, as shown in Figure 4J. It was also determined that the BAT and VAT weight decreased due to I3C administration (Figures 4K,L). Moreover, the serum FFA level was detected and the results revealed that serum FFA level reduced (Figure 4M). To illustrate the mechanism involved with the benefits of I3C on adipose tissue at the molecular level, the expression of lipid metabolism-related genes was detected by qRT-PCR assay. I3C administration increase the mRNA expression of genes involved with lipolysis (*Abhd5*) and fatty acid *β* oxidation (*Cpt1a*) as well as the white adipose tissue browning-related genes (*UCP1*, *Cidea*, *Prdm16*) in VAT (Figure 4N). Meanwhile, lipolysis (*Abhd5*) and thermogenesis-related genes (*UCP1*, *Prdm16*) in BAT were significantly increased after I3C administration (Figure 4O). Consequently, I3C administration improve HFD-induced expansion of adipose tissue and stimulate the expression of several genes involved with white adipose tissue browning in VAT and thermogenesis in BAT.

### 3.5 I3C improved HFD-induced liver steatosis and increased liver ILC1 as well as the related functional molecules

**Figure 5:**
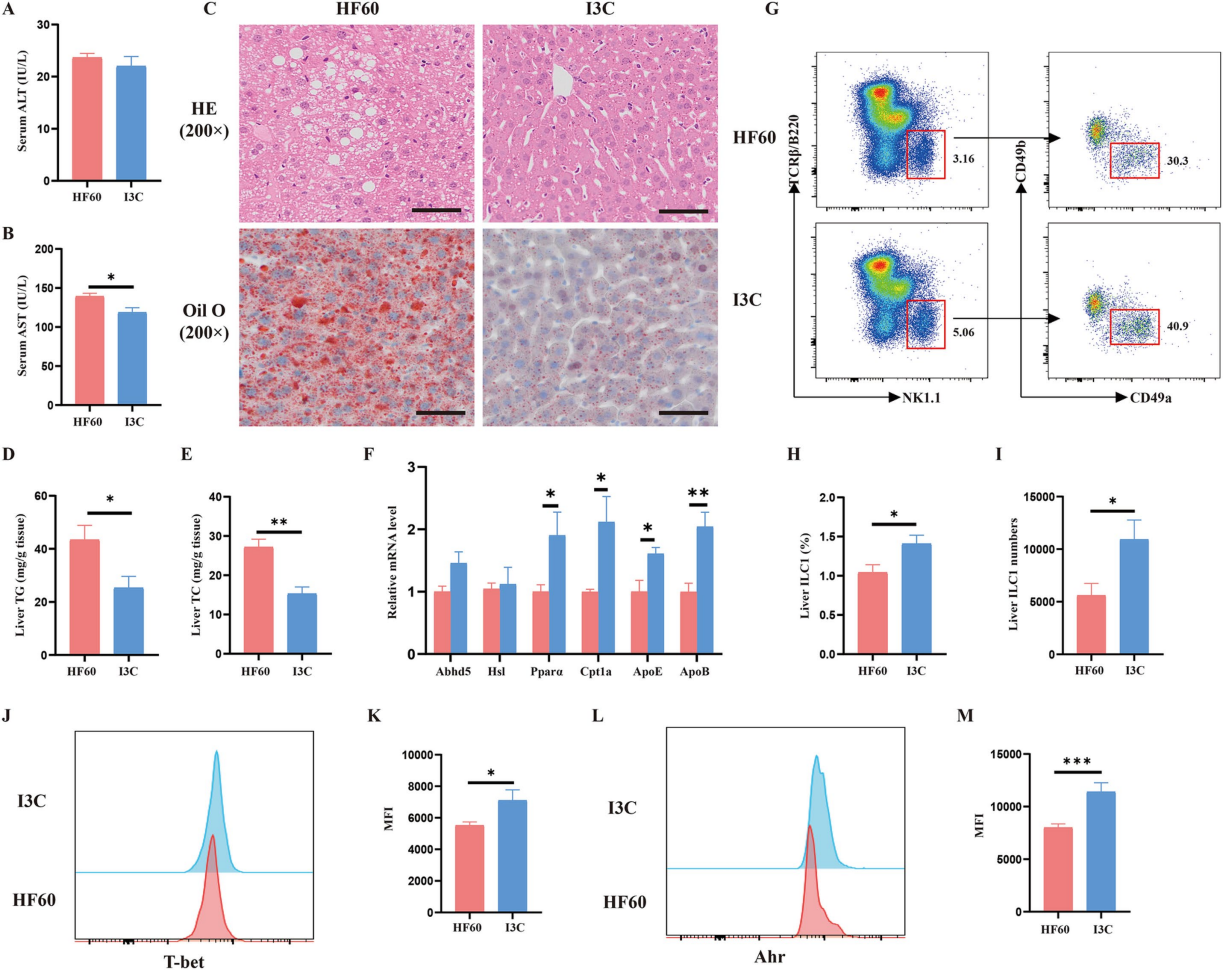
I3C improved HFD-induced liver steatosis and increased liver ILC1 as well as the related functional molecules. **(A)** Biochemical measurements of Alanine aminotransferase (ALT); **(B)** Biochemical measurements of Aspartate aminotransferase (AST); **(C)** Representative photographs of the liver sections with H&E (Up), Oil Red O (Down) staining. Scale bar: 50 μm; ×200 magnification; **(D,E)** The liver TG and TC levels were measured with the corresponding assay kits; **(F)** The mRNA expressions of the liver which related to lipid metabolism detected by qRT-PCR; **(G)** Representative flow cytometry of liver ILC1 cells (CD45^+^TCRβ^−^B220^−^NK1.1^+^CD49a^+^CD49b^−^); The percentage **(H)** and absolute numbers **(I)** of ILC1 in CD45^+^ lymphocytes; **(J,K)** Representative flow cytometry and MFI of T-bet in liver ILC1; **(L,M)** Representative flow cytometry and MFI of AhR in liver ILC1; *p < 0.05, **p < 0.01.

The effects of I3C on HFD-induced liver steatosis and the effect of liver ILC1 were additionally verified. As compared to the HF60 group, I3C produced no notable effect on serum ALT level whereas led to a reduced serum AST level (Figures 5A,B). At the same time, we found that I3C administration improved hepatic steatosis and reduced macro-vesicular lipid vacuolation as assessed by H&E and Oil Red O assays (Figure 5C). Meanwhile, the TG and TC levels in liver were significantly decreased as compared to HF60 group mice (Figures 5D,E). To illustrate the mechanism/effect of I3C on liver steatosis on the molecular level, the mRNA expression of lipid metabolism-related genes was analyzed. I3C dominantly increased the mRNA expression of genes involved with lipolysis (*Abhd5*) and fatty acid *β* oxidation (*Cpt1a, PPARα*). It also increased the expression of lipid transport-related genes (*ApoE, ApoB*; Figure 5F). Meanwhile, as compared to the HFD group mice, I3C administration tended to obviously increase the frequency and absolute number of the liver ILC1 (Figures 5G–I). We also revealed the expression level of liver ILC1 differentiation associated with transcription factors T-bet and AhR. The obtained results proved I3C to be able to increase the MFI of T-bet and AhR (Figures 5J–M). In summary, I3C administration improved HFD-induced liver steatosis and increased liver ILC1 as well as the related functional molecules.

### 3.6 I3C binds to AhR and regulates the AhR activation-related genes expression in VAT, BAT and liver

**Figure 6:**
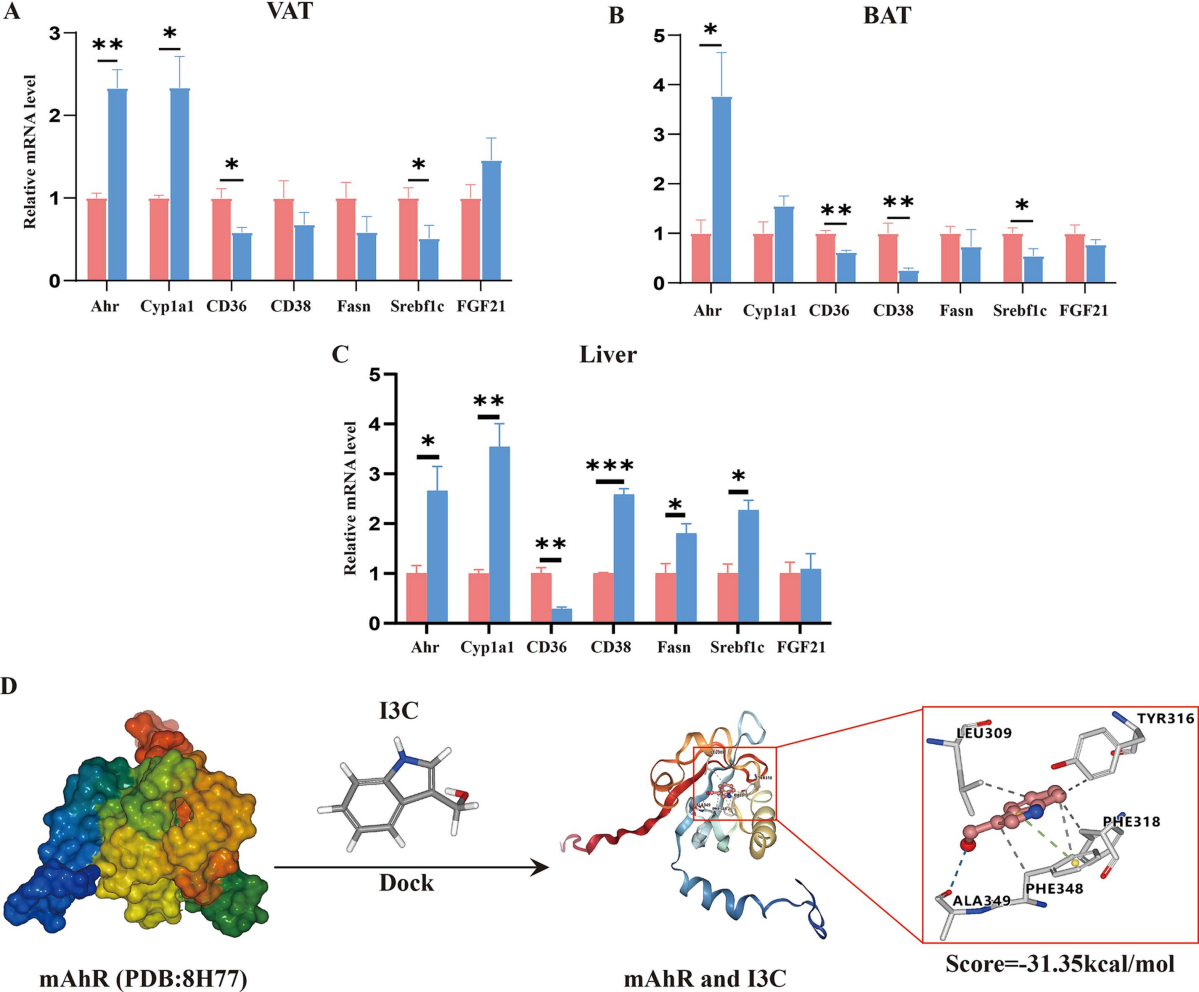
I3C binds to AhR and regulates the AhR activation-related genes expression in VAT, BAT and liver. **(A)** The mRNA expressions of AhR activation related genes in VAT were detected by qRT-PCR; **(B)** The mRNA expressions of AhR activation related genes in BAT were detected by qRT-PCR; **(C)** The mRNA expressionsof AhR activation related genes in liver were detected by qRT-PCR; **(D)** I3C and AhR molecular docking fitting model diagram, hydrogen bond interaction (blue line), hydrophobic interactions (gray line), *Π*-cation interaction (green line); *p < 0.05, **p < 0.01, ***p < 0.001.

To illustrate whether the protective effect of I3C was associated with AhR activation, the molecular docking and AhR activation-related genes expression were investigated. Initially, we examined the expressions of AhR-activation-related genes in VAT, BAT and liver by qRT-PCR assay, correspondingly. The results showed that the expressions of *AhR* in VAT and BAT as well as *Cyp1a1* in VAT were significantly increased (Figures 6A,B). Meanwhile, the expressions of *CD36* and *Srebf1* in VAT and BAT as well as *CD38* in BAT were also clearly decreased in I3C group mice as compared to the HF60 group (Figures 6A,B). Furthermore, comparison with the HF60 group, proved I3C to also significantly increase the relative mRNA expressions of *AhR* and *Cyp1a1* in liver (Figure 6C). Meanwhile, *CD36* mRNA expression level in liver was also obviously decreased in I3C group mice as compared to the one in HF60 group mice, whereas *Srebf1* and *CD38* mRNA expressions were significantly increased (Figure 6C). Consequently, I3C administration can active AhR-related genes in VAT, BAT and liver. To further clarify the potential interactions between I3C and mouse AhR crystal structure (PDB ID:8H77), the molecular virtual docking validation was performed. We found I3C to form a hydrogen-bond interaction with the residue ALA349 and hydrophobic interactions with the residues LEU309, TYR316, PHE318 as well as the *Π*-cation interaction with the residue PHE348, respectively. The conformational score was −31.35 kcal/mol (Figure 6D). In summary, I3C can bind to AhR directly and regulate AhR activation-related genes expression in VAT, BAT and liver.

### 3.7 I3C improved liver steatosis and reversed the decreased liver ILC1 induced by HFD in an AhR-dependent manner

**Figure 7:**
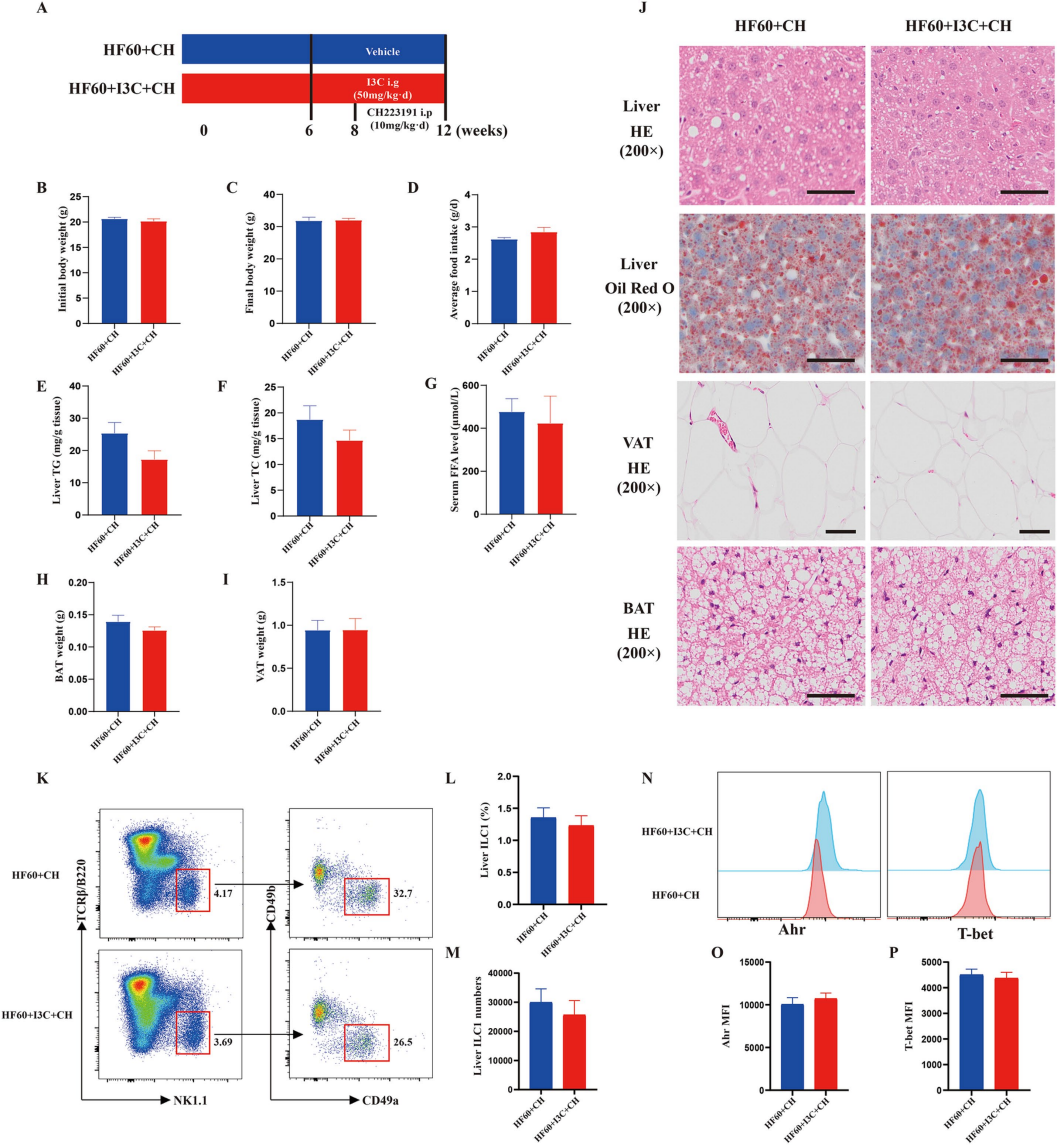
I3C improved liver steatosis and reversed the decreased liver ILC1 induced by HFD in an AhR-dependent manner. **(A)** Schematic diagram illustrating that 8-week-old male C57BL/6-mice were fed a HFD diet for 12 weeks, and I3C was given with the dose of 50 mg/kg/day intragastrically for 6 weeks, and CH223191 was given with the dose of 10 mg/kg/day Intraperitonealy for 4 weeks; **(B)** Initial body weight of mice; **(C)** Final body weight of mice; **(D)** Average daily food intake per mouse; **(E,F)** The liver TG and TC levels were measured with the corresponding assay kits; **(G)** The serum FFA level of mice; **(H)** Weight of VAT; **(I)** Weight of BAT; **(J)** Representative photographs of the liver section with H&E and Oil Red O staining and representative photographs of the VAT, BAT sections with H&E staining. Scale bar: 50 μm; ×200 magnification. **(K)** Representative flow cytometry of liver ILC1 cells (CD45^+^TCRβ^−^B220^−^NK1.1^+^CD49a^+^CD49b^−^); The percentage **(L)** and absolute numbers **(M)** of ILC1 in CD45^+^ lymphocytes; **(N–P)** Representative flow cytometry and MFI of T-bet and AhR in liver ILC1.

To further investigate whether the protective effect of I3C was AhR-dependent, the mice were administrated CH223191 using an AhR inhibitor (Figure 7A). The protective effect of I3C in weight loss was notably abolished by the AhR inhibitor CH223191 (Figures 7B–D). Moreover, the effect of I3C supplement attenuated liver steatosis and the level of liver TG and TC as well as the lipid deposits morphologically by HE and Oil Red O staining could be inhibited by CH223191 (Figures 7E,F,J). The reversed effect of serum FFA levels, VAT weight, BAT weight by I3C treatment was also abrogated by CH223191, correspondingly. (Figures 7G–I). In addition, the CH223191 administration also counteracted the protective effects of I3C in VAT expansion and brown adipose tissue lipid deposition (Figure 7J). Notably, the effect of I3C on liver ILC1 (Figures 7K–M) and T-bet as well as AhR (Figures 7N–P) was abolished by CH223191. Thus, the obtained results show that the protective effect of the I3C against liver steatosis and reversed the decreased liver ILC1 induced by HFD in an AhR-dependent manner.

## 4 Discussion

MASLD is characterized by steatosis of hepatic parenchymal cells and the accumulation of excessive lipid in the liver (35, 36). According to the researches, high fat diet could increase the burden of liver lipid metabolism and the risk of MASLD (37). In recent years, MASLD imposes a major economic burden on society and is the fastest-growing cause of cirrhosis, liver failure and liver cancer (38). However, there are currently less pharmacological treatments (Rezdiffra) available through some drugs were published in NEJM and submitted to FDA. Experimental evidence in animals and humans connecting the intestinal microbiota to MASLD is widely available (39, 40). FMT from patients which had undergone Roux-en-Y gastric bypass surgery performed in germ-free mice resulted in significant loss of weight and fat mass as well as the hepatic insulin resistance (41). Thus, fecal microbiota transplantation holds potential as a promising option in the treatment of MASLD. However, the mechanism was unclear. The most interesting finding of this study was that FMT improves hepatic steatosis induced by HFD in a mouse model associated with liver ILC1 regulation and indole-3-carbinol level.

As we know, the liver is not only the largest metabolic organ involved in glucose and lipid metabolism (42) but also a well-recognized and complex immunological organ that contains numerous adaptive and innate immune cells (21). Emerging evidence indicated that liver immune cells participated in the liver lipid metabolism and MAFLD occurrence (43). Recent studies have identified that liver ILC1s played an essential role in liver virus infection, tumor development, liver injury and regeneration (29, 31). This study revealed liver ILC1 frequency to be negatively corrected with liver TC and TG level. In recent years, gut microbes dysbiosis is highly recognized as a key mechanism in the HFD induced regional immune dysregulation and occurrence of chronic metabolic diseases such as MASLD, diabetes through the “gut-liver axis” (44). In our study, we also found out that transplantation of the gut microbes of healthy mice could reduce the body weight and improve the liver steatosis induced by high-fat diet, which similar to previous conclusions (7, 8). Based on the obtained results, it was also proven that FMT could reverse the decreased liver ILC1 induced by high-fat diet. The results imply that FMT improves liver steatosis induced by HFD associated with liver ILC1 regulation. However, the mechanisms have not been well evaluated and clearly understood.

Several hypotheses have been proposed and one possibility is that imbalanced microbial metabolites regulate lipid metabolism and liver reginal immune function through the “gut-liver axis” (45). Previous studies supported an intimate relationship between microbiota-derived tryptophan (Trp) metabolites disorder, especially the decreased indole derivatives and the occurrence of metabolic diseases (46). In our study, we firstly proved FMT to be able to increase the expression of AhR of liver ILC1 in the HFD-fed mice. Thus, we suspected that FMT improves liver steatosis and increased liver ILC1 frequency which are associated with the indole derivatives. Indole-3-carbinol (I3C) was found in vegetables of the *Cruciferae* family such as broccoli, brussels sprouts, and cauliflower, or was produced by gut microbiota, which is an indole metabolite and may be a significant AhR ligand (47, 48). I3C is susceptible to oligomerization under acidic conditions approximating in gastric juice. And after ingestion, it is converted into several condensation products, such as 3,3-diindolylmethane (DIM), [2-(indol-3-ylmethyl)-indol-3-yl] indol-3-ylmethane (LTr1), indole[3,2b] carbazole (ICZ) and 1-(3-hydroxymethyl)-indolyl-3-indolylmethane (HI-IM). Recent years, I3C has received much attention as a promising preventive and treatment agent for various diseases such as cancers, diabetes and obesity (14–16, 49). In this study, we demonstrated that the I3C level was significantly decreased in HFD-fed mice and FMT could increase the serum level of I3C. And correlation analysis also proved the level of serum I3C to negatively correlate with the liver weight, TG and TC contents in the liver and positively correlated with liver ILC1 frequency of mice. Furthermore, we also found that I3C intervention mice had lower body weight, body fat ratio, AST level and liver TG and TC contents as compared to the high-fat diet induced mice. In addition, we found that the mRNA expressions of liver lipolysis gene abhd5 which encoding ATGL and fatty acid *β* oxidation genes Cpt1a and PPARα in liver were increased in the I3C intervention group, while the mRNA expression of fatty acid transport gene CD36 was decreased and ApoE, ApoB mRNA expressions in liver were significantly increased. Previous studies showed I3C to be a tryptophan-derived bacterial metabolite and possess potent anti-inflammatory, anti-oxidation and anti-cancer activity in numerous models (11, 43, 47). And, I3C improving glucose tolerance, reducing body weight gain and fat content as well as hyperlipidemia, inflammation was proved (16, 50). In this study, interestingly, we further found I3C can improve HFD-induced liver steatosis and increased liver ILC1 as well as the related functional molecules. Thus, we proposed that FMT improved hepatic steatosis and reversed decreased ILC1 induced by HFD associated with the level of microbial metabolites I3C. However, due to the limited bioavailability, whether the observed effects in our study are mediated by I3C itself or by its metabolites such as DIM, ICZ, LTr1, HI-IM should be additionally clarified.

Moreover, the molecular target of I3C biological activity in the lipid metabolism still has not been properly explored. Microbial metabolites are ligands for the host receptors such as the Aryl hydrocarbon receptor (AhR) (51). AhR is a nuclear receptor and transcription factor that widely exists in various tissues (especially intestine, liver and kidney) and cells of mammals (52). Being a ligand-dependent transcription factor, AhR belongs to the PER-ARNT-SIM subgroup of the basic helix–loop–helix (BHLH) protein superfamily (53), depending on a number of ligands. Ligands can be divided into endogenous and exogenous, the latter category including compounds synthesized by the body (e.g., apolipoprotein a4, tryptamine) and compounds produced by the gut flora (e.g., kynuretenic acid, indole and its derivatives), Exogenous involvement of dietary compounds (e.g., Quercetin, curcumin), environmental pollutants (e.g., Polyaromatic hydrocarbons, dioxins), drugs (e.g., Avitriptan, smetinib), and chemical synthetic compounds (e.g., SP600125, carbardine) (54). It was widely recognized that indole and indole derivatives act as the ligands of AhR (55). The AhR senses the signals generated by the environment, diet, microbes, and metabolism to initiate target genes transcription, thereby regulating numerous physiological and pathological processes (18). According to the studies, AhR activation can inhibit the saturated fatty acid synthesis in hepatocytes and attenuate inflammatory responses as well as the macrophage migration under lipid loading (56). The studies demonstrated Cyp1a1 to be a key biomarker of AhR activation. Meanwhile, CD36, CD38, Srebp-1c, Fasn, FGF21 were the downstream effector molecules of AhR (57). To sum it up, it still needs to be further explored whether AhR activation is the mechanism of I3C in improving HFD-induced lipid metabolism disorder. This study showed the mRNA expression level of the genes in liver, VAT and BAT tissues to be all changed after I3C intervention. Besides, the cryo-EM structures of the Hsp90-AhR-p23 complex and a group of conserved pocket inner residues in the AhR PAS-B domain were clarified in 2023 (34). In our current study we found I3C could bind to residues of ALA349, PHE348, LEU309, TYR316, PHE318 on AhR through hydrogen bonds, *Π* bonds, hydrophobic bonds. And we also confirmed that I3C improved liver steatosis and increased liver ILC1 frequency induced by HFD were notably abolished by CH-221391, a potent AhR antagonist.

In conclusion, our study suggests that healthy gut microbiota transplantation ameliorated high-fat diet-induced liver steatosis and reversed decreased liver ILC1 induced by HFD associated with the level of indole-3-carbinol and AhR activation. Furthermore, I3C regulated the changes of body composition, lipid metabolism and thermogenesis-related genes in VAT and BAT and improved liver steatosis induced by HFD as well as increased liver ILC1. Moreover, as for the molecular target, I3C could bind to many residues of AhR through hydrogen bonds, Π bonds, hydrophobic bonds. Our data, therefore, highlight the potential treatment value and mechanism as well as the theoretical basis of FMT and microbiota-derived I3C in the contribution of controlling hepatic steatosis and MASLD.

## Data Availability

The datasets presented in this study can be found in online repositories. The names of the repository/repositories and accession number(s) can be found in the article/supplementary material.

## Footnotes

^1^ https://cloud.yinfotek.com

^2^ http://www.rcsb.org

## Glossary

AhR: Aryl hydrocarbon receptor
AUC: Area under the curve
ALT: Alanine aminotransferase
AST: Aspartate aminotransferase
BAT: Brown adipose tissue
FMT: Fecal microbiota transplantation
FFAs: Free fatty acids
Glu: Glucose
HFD: High-fat diet
I3C: Indole-3-carbinol
ICZ: indole[3,2b] carbazole
HDL: High density lipoprotein-cholesterol
HE: Hematoxylin and eosin
LDL: Low-density lipoprotein-cholesterol
OGTT: Oral glucose tolerance tests
Oil O: Oil red o staining
qRT-PCR: Quantitative Real-Time Polymerase Chain Reaction
TC: Total cholesterol
TG: Triglyceride
VAT: Visceral adipose tissue
HI-IM: 1-(3-hydroxymethyl)-indolyl-3-indolylmethane
LTr1: [2-(indol-3-ylmethyl)-indol-3-yl] indol-3-ylmethane
DIM: 3,3-diindolylmethane
